# Risk factors of breast cancer among patients in a tertiary care hospitals in Afghanistan: a case control study

**DOI:** 10.1186/s12885-021-07798-5

**Published:** 2021-01-15

**Authors:** Zekrullah Baset, Jamshid Abdul-Ghafar, Yasmin Nadeem Parpio, Ahmed Maseh Haidary

**Affiliations:** 1 Department of Pathology and Clinical Laboratory, French Medical Institute for Mothers and Children, Kabul, Afghanistan; 2 School of Nursing and Midwifery, Aga Khan University, Karachi, Pakistan

**Keywords:** Risk factors, Women, Breast cancer, Afghanistan

## Abstract

**Background:**

Breast cancer is the second most common causes of women’s death, worldwide. Data on risk factors associated with female breast cancer in the Afghan population is very limited. The aim of our study was to identifying risk factor associated with female breast cancer in Afghanistan.

**Methods:**

A retrospective case-control study was conducted with inclusion of 201 cases and 201 controls. Patient information was collected by interviewing the patient through a structured questionnaire. Histopathological information was collected from the hospital integrated laboratory management system. The data was analyzed by using logistic regression with univariate and multivariable analyses to determine the association between breast cancer and predictors.

**Results:**

The results of the current study showed that factors such as: age (OR = 1.02; 95%CI: 0.99–1.04; *p*-0.148); age at menarche (OR = 0.83; 95%CI: 0.72–0.92; *p*-0.008); age at first baby (OR = 1.14; 95%CI: 1.07–1.20; *p*- < 0.001); illiteracy (OR = 1.93; 95%CI: 1.16–3.22; *p*-0.011); smoking (OR = 2.01; 95%CI: 1.01–3.99; *p*-0.04) and family history of cancer (OR = 1.98; 95%CI: 1.18–3.32; *p*-0.009) were significantly associated with breast cancer. However, our study did not demonstrate any statistically significant correlation between breast cancer and some of the predictors that were previously highlighted in literature, such as: marital status, Body Mass Index (BMI), use of hormonal contraceptive, breastfeeding and exercise.

**Conclusion:**

Our study demonstrated that age at menarche, and age at first baby birth, illiteracy, smoking and family history of cancer were significant risk factors associated with development of breast cancer among women in Afghanistan. Health education of women regarding aforementioned predisposing factors are therefore, expected to be valuable in decreasing the burden of breast cancer with reduction of its burden on the healthcare system in Afghanistan.

## Background

Cancer is the world’s leading cause of mortality. World Health Organization (WHO) reported that 7.6 million people died of cancer in 2005 and it is predicted that 84 million people will die in the next 10 years [1]. Like other malignant disorders, the genomic machinery that directly regulates cellular proliferation, are affected during the pathogenesis of breast cancer [2].

Breast cancer is a global health problem, affecting breast parenchyma, with a global incidence of 1.7 million new cases per year [3]. Constituting 23% of all cancers around the world, breast cancer is the most common malignancy in women, causing most of the cancer related death in women, being the second most common cause of women death worldwide [4, 5]. According to latest reports, breast cancer is a rapidly growing disease in South America, Africa and Asia-pacific, thus warranting the need for early detection of breast cancer for reducing the mortality [6]. Although the recent figures from most of the Asian countries demonstrated that female breast cancer was the most common malignancy in most of the Asian countries, still in some regions of the developing world, it remains the second most common malignancy followed by uterine cancer. Alarmingly, around 60% of women’s death are due to breast cancer in many of the developing countries [3]. On the other hand, although, considering the available reports from underdeveloped and developing countries in Asia, the projected incidence of female breast cancer was lower if compared to the Western countries, still the mortality related to breast cancer was higher than the Western developed world [6]. The reason behind the higher mortality could be the scarce availability of modern diagnostic as well as therapeutic interventional facilities in underdeveloped and developing regions of Asia.

In Pakistan, the incidence of female breast cancer was reported to be 50.1 per 100,000 making it the most common malignancy in Pakistani women [6]. Although incidence rate of female breast cancer is not available for whole Indian population, according to female breast cancer registry, the incidence of breast cancer in three important regions of India, that are Bombay, Bangalore and Madras, was reported to be 25.6, 15.8 and 20.1, respectively, per 100,000 female population, in 1985 [7]. In Iran too, the female breast cancer ranked to be the first and the most common malignancy among women, constituting 21.4% of all female malignancies. Accordingly, in Tehran alone, which is the capital city of Iran, female breast cancer was one of the most common malignancies in women compared to other malignancies with an incident rate of 22.4 per 100,000 in 1998 [8]. While over 90% of symptomatic breast lesions are benign, breast cancer constitutes a heterogeneous group of malignant disorders with variable clinical features, histological characteristics and variable therapeutic outcomes [9, 10]. Breast cancer is primarily classified on the basis of histological appearance, either into lesions that originate from the ductal epithelium (inner lining) or the lobular epithelium, which are the conduit of milk to ducts [11]. WHO has classified the breast cancers into 21 distinctive histological types, based on cell morphology, growth and architectural patterns [12]. The two most common among all breast cancers are the Invasive Ductal Carcinoma (IDC) and Invasive Lobular Carcinoma (ILC), accounting for up to 75 and 15%, of incidence, respectively [13]. The remaining 10% of malignancies related to female breast include rare histologic types of mucinous, metaplastic, inflammatory, medullary, and papillary carcinomas [14]. Worldwide, a large number of breast lesions are diagnosed due to widespread usage of screening breast lesions by mammography and latest imaging technologies in the pre-clinical phase [15].

There are some established risk factors associated with female breast cancer, which can be divided in two main categories, i.e. non-modifiable risk factors including age, sex, family history of breast cancer, proliferative breast disease and the modifiable risk factors including exposure to estrogen, weight, alcohol consumption, smoking, physical activity, diet, anxiety and stress and exposure to oral contraceptive [16].

According to the estimations by neighboring countries, in Afghanistan, death due to cancer before the age of 75 years accounts for 11.4% of mortality in males and 10.2% in females [17]. Considering the available data from neighboring countries, although in Afghanistan the incidence of breast cancer is not high, due to scarcity of appropriate health services the mortality rate is reported to be equal to or even more than Western countries [18].

There is a scarcity of research regarding breast cancer in Afghanistan and the available estimates are mostly dependent on data generated from neighboring countries. We conduct this study to estimate the risk factors associated with breast cancer in Afghan female population.

## Methods

### Study design

For this research, a retrospective case-control study design was used. This study compared the female breast cancer (outcome of interest, cases) with non-breast cancer (controls), and observed the association of potential risk factors present in each group.

### Study population

The study population consisted of 201 women with breast cancer aged 30 years and older, paired by age, admitted between January 2018 to December 2019, at French Medical Institute for Mothers and Children (FMIC) and Jamhoriat Tertiary Care Hospital (JTCH), two of the reference institutions for histopathology and oncology in Kabul, Afghanistan. Out of 201 cases of breast cancer, 178 cases were invasive ducal carcinoma, 6 cases were invasive lobular carcinoma, 4 cases were medullary carcinoma, 1 case was metaplastic carcinoma and 12 cases was other malignant breast cancer.

### Sample size

For sample size calculation we used OpenEpi (open source epidemiologic statistics for public health) statistical software. Taking the confidence interval of 95%, power of the study 80% and case and control ratio as (1:1) by taking odds ratio of 2.78 of parity the minimum sample size for case and control was calculated 382 [19]. Taking non-respondents, errors, withdrawal and missing data into consideration, the total sample size has been inflated by 5%, the final sample size calculated 201 case and 201 controls.

### Selection of cases and controls

Cases were defined as female participants with breast cancer diagnosed by histopathologic examination and age ≥ 30 years. The control group included the women without breast cancer with the same age of the case. The controls were recruited from the same hospital and were confirmed for the disease status by the same histopathological examination as for cases.

### Data collection

All cases were collected mainly from department of oncology, JTCH and form Integrated Laboratory Management System (ILMS) of FMIC. A pre-test was done before collection of the data. Patients were called to fill the questionnaire on the day of collecting the report. A written consent was taken from patients. The interview was conducted mainly by the female co-investigator. All items included in the questionnaire had short answer or were single choice questions. Data for control group was collected randomly from department of obstetrics & gynecology, FMIC, department of pathology & clinical laboratory and Isteqlal tertiary care hospital (ITCH) from those patients who were admitted with no history of breast neoplastic disease.

### Data collection tool

Data was collected using the tool developed by the principle investigator. The tool was developed in English language and translated to the local Farsi/Dari language. Before using the tool, it was sent to five experts to check the clarity, relevance and context of the tool on scale of 1–4. The content validity index for clarity and relevance was calculated, which was found to be 0.92 and 0.94, respectively. For estimation of the reliability of the study, Cronbach alpha test was conducted, the result was 0.83. The tool was pretest upon 10% of population, some changes were made based on pretesting results. Pretest data was not included in the study. Each interview was taken in a comfortable place and lasted for15–20 min in Isteqlal hospital, the data was collected by a female data collector who was well trained by the principle investigator. Whereas, in other centers (FMIC, JTCH), data were collected by the principal investigator. Variables such as use of oral contraceptive, history of breast feeding, smoking, BMI, exercise, educational status, age, pregnancy history, family history of cancer, age at menarche, marital status were assessed with the help of a questionnaire.

### Data management and quality control

In order to ensure quality of data collection, the data collector was properly trained by principal investigator. However, the questionnaire was self-administered tool but data was collected by interview to help reduce errors. For first few days the data collector had collected data under the supervision of the principal investigator to fully and accurately understand the process of data collection. Field editing and office editing was done by the principle investigator to identify any missing or wrong entries on daily basis. Data was entered into Statistical Package for Social Sciences (SPSS, version 25.0) by double entry strategy. Hard copy of data was kept under lock and key and soft copy of data was kept on password protected personal computer to which only principle investigator had access. The data has been already shared with committee members.

### Data analysis

Mean and standard deviation (SD) were estimated for continuous/discrete variables. For categorical variables, frequency and proportions were calculated. Logistic regression was applied to measure the association amongst independent variables and dependent variables. The logistic regression was carried out in two steps. In the first step association between breast cancer and each independent variable was examined while the dependent variable and the odds ratios were estimated. In the second step, all the variables which were found significant at the univariate level (*p*-value ≤0.25), were considered for multivariable model. The multivariate analysis of risk factors was calculated through enter method for the construction of model and controlling the confounders in the study. Odds ratio (OR) and 95% Confidence Interval (CI) were reported to determine the association between breast cancer and risk factors.

## Results

The descriptive statistics for case and control as continuous variable are listed in Table 1. Similarly, the descriptive statistics for categorical variables are shown in Table 2.

**Table 1:** Descriptive statistics of breast cancer cases (*n* = 201) and controls (*n* = 201) in Afghanistan for continuous variables

	Cases (***n*** = 201)	Controls (***n*** = 201)
Variables	Mean (SD ^a^)	Mean (SD)
Age in years	45.8 (10.5)	41.7 (10.1)
BMI ^a^	26.3 (5.3)	26.7 (5.3)
Age at first baby birth	23.1 (5.3)	20.7 (3.9)
Age at menarche	13.3 (2.1)	13.9 (1.6)

^a^*SD* Standard Deviation, *BMI* Body Mass Index

**Table 2:** Descriptive statistics of breast cancer cases (*n* = 201) and controls (*n* = 201) in Afghanistan for categorical variables

Variables	Cases (***n*** = 201) n %	Controls (***n*** = 201) n %
Educational status		
Illiterate	121 60.2	89 44.3
Literate	80 39.8	112 55.7
Marital status		
Single	25 12.4	27 13.4
Married	176 87.6	174 86.6
Family history of breast cancer		
No	115 57.2	153 76.1
Yes	86 42.8	48 23.9
History of pregnancy		
Yes	163 81.1	161 80.1
No	38 18.9	40 19.9
History of contraceptive use		
No	145 72.1	145 72.1
Yes	56 27.9	56 27.9
History of breast feeding		
Yes	150 74.6	149 74.1
No	51 25.4	52 25.9
Exercise		
No	159 79.1	154 76.6
Yes	42 20.9	47 23.4
Smoking status		
No	156 77.6	178 88.6
Yes	45 22.4	23 11.4

### Descriptive statistics of continuous variables

Table 1 describes the distribution of continuous variables of age, Body Mass Index (BMI), age at first baby and age at menarche for both cases and controls. The age of the participants was ≥30 (measured in years). The mean age (±SD) for cases and controls was 45.8 (±10.5) and 41.7 (±10.14), respectively. However, the mean BMI for cases was 26.3 (±5.3), while for controls it was 26.7 (±5.3). The mean age at first baby’s birth in cases was 23.1 (±5.3) while the mean age at first baby for controls was 20.7 (±3.9). Among cases, the mean age at menarche was 13.3 (±2.1) and for control it was 13.9 (±1.65).

### Descriptive statistic of cases and controls on categorical variables

Table 2 explains the descriptive statistics on categorical variables. Among cases, 121 (60.2%) were illiterate whereas 89 (44.3%) of the controls were illiterate. 176 (87.6%) women from cases were married, while 174 (86.6%) women from controls were married. Family history of cancer was present in 57.2% of case and in 76.1% of control groups. Out of 201 cases, 150 (74.6%) breastfeed their children, while 149 (74.1%) of the controls had history of breastfeeding. The use of contraceptive was similar in both cases and controls 56 (27.9%). In cases, 45 (22.4%) were smokers, the frequency of smoking among controls was 23 (11.4%). Only 42 (20.9%) women from case group were physically active and doing regular exercise, while it was 47 (23.4%) in control group women.

### Test statistics for normality check of continuous variables

Table 3, after descriptive statistics, we evaluated the normality for continuous variables. There were four continuous variables in this dataset. Since the sample size is more than 50, so we looked for Kolmogorov-Smirnov instead of Shapiro test. Initially we looked at histograms, the graphical representation showed the variables are not symmetrical. Then we looked at the *p*-values of Kolmogorov-Smirnov test. Since *p*-values were less than alpha so we reject null hypothesis and conclude that the data is not normally distributed.

**Table 3:** Test statistics for normality check of continuous variables

Variables	Test statistics	***p***-value
Age	0.134	< 0.001
Age at the first baby	0.118	< 0.001
Age at menarche	0.144	< 0.001
BMI^a^	0.064	0.003

^a^*BMI* Body Mass Index

### Logistic regression

Inferential statistics, i.e. logistic regression, was applied to identify the association among dependent variables and independent variables. Logistic regression is considered the best for describing the association between a categorical dependent variable and one or more continuous or categorical independent variables. The logistic regression was carried out in two steps. In the first step association between each independent variable was examined with the dependent variable and the odds ratios and their 95% CI were computed. All the variables which were found significant at the univariate level (*p*-value≤0.25), were considered for multivariable model.

### Univariate analysis

The unadjusted OR and their 95% CIs are reported in Table 4. The variables which were found to be significant were age, age at first period, age at first baby birth, education, family history of breast cancer, and smoking status. The other variables which were added in logistic regression model at univariate level were marital status, BMI, history of pregnancy, history of contraceptive, status of breast feeding and exercise did not show significant association.

**Table 4:** Univariate logistic regression of socio-demographic and potential risk factors associated with breast cancer in Afghanistan (*n* = 402)

Variables	UAO^**a**^	95% C.I	***p***- value
Age	1.04	(1.02–1.06)	< 0.001
Educational Status			
Literate	1.90	(1.28–2.83)	< 0.001
Illiterate	1		
Marital status			
Single	1		
Married	0.92	(0.51–1.64)	0.77
BMI^a^	0.98	(0.95–1.02)	0.38
Family history of breast cancer			
Yes	1		
No	2.38	(1.55–3.65)	< 0.001
History of pregnancy			
Yes	1		
No	0.94	(0.57–1.54)	0.801
Age at first period	0.85	(0.76–0.95)	0.003
Age at first baby birth	1.12	(1.06–1.18)	< 0.001
Use of contraceptive			
Yes	1		
No	1.000	(0.65–1.55)	1.000
Status of breast feeding			
Yes	1		
No	0.97	(0.62–1.53)	0.909
Exercise			
VYes	1.16	(0.72–1.85)	0.55
No	1		
Status of smoking			
Yes	1		
No	2.23	(1.29–3.9)	0.004

^a^*UOR* Unadjusted Odds Ratio, *BMI* Body Mass Index

The patient age was found to be significantly associated with breast cancer. With one-unit increase in age, the risk of breast cancer also increased as evident by (OR = 1.04, 95% CI: 1.02–1.06, *p*-value< 0.001). Moreover, an association between level of education and breast cancer was also statistically significant (*p*-value< 0.001). The risk of breast cancer was found to be 1.9 times to the risk of breast cancer among illiterate cohort (OR = 1.9, 95%CI: 1.28–2.83). Age at first period was also significantly associated with breast cancer. It was observed that with 0.85-fold decrease in age at menarche, the risk of cancer is also decreasing (OR = 0.85, 95%CI: 0.76–0.95, *p*-value = 0.003).

Likewise, a significant association was also observed between smoking and breast cancer (*p*-value = 0.004); among the participants who were active smoker the risk of breast cancer is 2.23 times more than non-smokers (OR = 2.23, 95% CI: 1.29–3.9, *p*-value = 0.004). Like smoking, family history of cancer was significantly associated with breast cancer, it suggests those who has family history of cancer are 2.38 times more prone to breast cancer; as compared to those who have no family history of cancer (OR = 2.38, 95%CI: 1.55–3.65, *p*-value< 0.001). Furthermore, the age at first baby birth is significantly associated with breast cancer, it showed that by one-year increase in age the risk of breast cancer also increases (OR = 1.12, 95%CI: 1.06–1.18, *p*-value< 0.001).

### Multivariate analysis

As illustrated in Table 5, the multi-variable model includes five variables which came out to have significant association at the multivariate level (*p*-value< 0.05). The final model, variable such as age at first period, age at first baby birth, education level, smoking and family history of breast cancer found to have statistically significant association with female breast cancer. However, variables such as marital status, BMI, history of pregnancy, contraceptive, breast feeding and exercise were not statistically significant with female breast cancer.

**Table 5:** Multivariate logistic regression for risk factors associated with breast cancer in Afghanistan (*n* = 402)

Characteristics	AOR^**a**^	95% CI	***p***-value
Age	1.02	(0.99–1.04)	0.148
Age at menarche	0.83	(0.72–0.92)	0.008
Age at first baby birth (in year)	1.14	(1.07–1.20)	< 0.001
Educational Status			
Literate	1.93	(1.16–3.22)	0.011
Illiterate	1		
Smoking status			
Yes	1		
No	2.01	(1.01–3.99)	0.04
Family history of breast cancer			
Yes	1		
No	1.98	(1.18–3.32)	0.009

^a^*AOR* Adjusted Odds Ratio

Age which was significantly associated (*p*-value< 0.001) with breast cancer in univariate level; however, it is not significant in multivariate analysis (*p*-value = 0.148). Age at first period (menarche) is significantly associated with breast cancer (OR = 0.83, 95% CI: 0.99–1.04, *p*-value 0.008). As age at first period increases the odds of breast cancer was 0.83 times; conversely, with increase in age at first baby birth, the risk of breast cancer increases (OR = 1.14, 95%CI: 1.07–1.20, *p*-value< 0.001).

Family history of cancer was found to be an important risk factor for breast cancer. The odds of having a family history of breast cancer is 2.2 times higher among cases as compared to controls (OR = 2.2, 95% CI: 1.4–3.5). Similarly, smoking was found to be a risk factor for developing breast cancer as evident by (OR = 2.01, 95% CI: 1.01–3.99). Likewise, education level was found to be a significant factor for case. The odds of being illiterate was 1.93 in cases as compared to odds of being illiterate among controls (OR = 1.93, 95%CI: 1.16–3.22, *p*-value = 0.011).

### Model fitness and significance

The findings of the multivariate analysis found overall model found to be significant as evident by significant *p*-value of less than 0.001 in omnibus test of model coefficient. We have sufficient evidence to conclude that age, age at first baby, age at menarche, illiteracy, smoking status and family history of breast cancer were statistically associated with breast cancer in multivariate adjusted model. Finally, the model explained 21.6% of the variability in breast cancer is explained by all the independent variables present in final model as evident by Nagelkerke R square value. Goodness for fit test, the Hosmer and Lemeshow test statistics was 8.375 with *p*-value of 0.398 which shows that fail to reject null hypothesis and conclude that the model was well fit to the data.

## Discussion

The study aimed to identify the factors associated with development of female breast cancer among Afghan population. The current research demonstrated a significant association between age, age at menarche, age at first baby birth, illiteracy, smoking and family history of cancer with breast cancer among females in Afghanistan. The study did not reveal any statistically significant associations between breast cancer and previously highlighted predictors of the disease, such as: marital status, BMI, use of hormonal contraceptive, breastfeeding and exercise, probably due to sampling error and low power of the research.

Age was not significantly associated with breast cancer, when considering multivariate model but due to clinical significance we preserved this variable in the final model. Univariate analysis revealed that as age increased by one unit (year), the risk of breast cancer also increased. The findings of our study were comparable with the previous studies. As the patient age increased, the incidence of breast cancer increased. Younger age at the time of diagnosis was linked with high mortality [20]. A study conducted in Poland demonstrated findings that were similar to our study. They reported that breast cancer was frequently diagnosed around menopause and significantly less common below the age of 45 years [21]. Similarly a study conducted by Helena D et al., demonstrated that age had significant association with development of breast cancer [22]. As the women age, damages occur to the ductal epithelial cells of mammary glands, thus increasing the chance for neoplastic transformation. The plausible reason for this relationship could be a prolonged exposure to estrogen which is an independent risk factor for breast cancer development; furthermore, with aging, depending on lifestyle, the possibility of sustaining cellular damage via exposure to radiation and other environmental oncogenic risk factors can also be deemed accountable to affect the genomic machinery [23].

The current study revealed a significant association between early age at menarche and development of breast cancer. As age at first period increased, the risk of breast cancer development was shown to reduce by 0.83 times. These findings were consistent to the findings of a study conducted by Monteiro DLM et al., reported that early menarche was a strong risk factor for breast cancer [24]. Similarly, in another study, a strong association was demonstrated between early age at menarche and ILC of breast [25]. Data from the study conducted by Bhupathi S et al. also suggested that there was a significant association between early age at first menarche and breast cancer [19]. Furthermore, a study conducted in Morocco revealed a significant association of early menarche with breast cancer in univariate level of logistic regression analysis [26]. Estrogen and progesterone are the hormones produced by ovaries and it was frequently reported that these hormones were stimulators for breast cancer; in early menarche, women are exposed earlier to these two hormones, thus, the risk of breast cancer increases [27]. It was also reported that early menarche was associated with ILC of breast which are estrogen receptor positive tumors [27, 28]. Surprisingly, a study conducted in Turkey demonstrated no correlation of statistical significance between early menarche and breast cancer [29]. On the contrary, there were no available data in the literature that would show a negative association between early age at menarche and development of breast cancer.

The present study suggested that late age at first baby born was a risk factor for developing breast cancers. As the age at birth of first baby increased, the risk of breast cancer also increased by 1.14 times per year. Monteiro DLM et al. also demonstrated that age at first pregnancy was significantly associated with development of breast cancer [24]. Another study demonstrated that first pregnancy after age 35 increased the risk of breast cancer [30]. The possible reason could be the activation of JAK-STAT5 signaling pathway which in its activated form, the pSTAT5 has been proven to be a pro-survival promotor protein for ductal cells. The mentioned activated protein increases during pregnancy and lactation, thus, those proteins protect the ductal cells from mutations. It has been shown that during pregnancy breast cells multiply less, leading to lesser development of tumor which could be a protective factor. The reason behind increased risk of breast cancer with first pregnancy at age 35 may be breast tissue which are responsible of carrying cluster of cells with cancer causing mutation [30].

The literacy was associated with breast cancer in the current study. A study conducted in Iran on population of 1,477,045, also had similar findings, in which, out of 770 women who had breast cancer 287 (37.3%) were illiterate [31]. A study done in Bangui of Central Africa showed that lack of education was significantly associated with breast cancer (*p*-value< 0.001) [32]. Literacy was a protective factor for breast cancer according to Harirchi I et al. [31].

The present study provided an evidence of a significant association between smoking and development of breast cancer where the smokers were 2.01 times more prone to developed breast cancer as compared to control group. Consistent with current study, a research done by Catsburg C et al. depicted a strong association between smoking and breast cancer [33]. Moreover, most of literature linked smoking with higher risk of breast cancer [34]. Furthermore, a study showed significant association between smoking and breast cancer, in those female who do not used alcohol [35]. As per our observation, chillum (pipe smoking) is very common among women in Afghanistan. The most probable reason could be the uptake of tobacco carcinogens by breast ductal cells including N-nitrosamines, polycyclic aromatic hydrocarbons and aromatic amines, that are mostly involved in mutation of p53 gene [34].

The findings of our study showed that positive family history of cancer was significantly associated with breast cancer, with the odds of having a positive family history of cancer being 2.2 times higher among cases as compared to controls, which was similar with the findings of a study done by Laamiri FZ et al. who demonstrated that positive family history in immediate relatives was significantly associated with breast cancer, however, they didn’t report any association between breast cancer and positive family history in second degree relatives [26]. Likewise, in another study conducted in Iraq, the Iraqi patients who developed breast cancer had 30% positive family history of cancer. Among those, 18.5% had family history of breast cancer [36].

Similar to our study, a previous study conducted in Central Africa found that positive family history was a strong risk factor for developing of breast cancer [32]. The major reason behind this association might be inherited gene mutation such as Breast Cancer Gene 1 (BRCA1) or Breast Cancer Gene 2 (BRCA2) mutations. On the other hand, some literature also suggested the checkpoint kinase 2 (CHEK2, Chk2) gene role for developing breast cancer [37, 38]. Contrary to the findings of the present study, Monteiro DLM et al. reported no relationship between positive family history of cancer, use of hormonal contraceptive, use of alcohol, ethnicity, tobacco, and breast cancer [24].

Our study was conducted in multiple tertiary care hospitals which increased the probability of representing Afghanistan’s whole population. This research was made with an effort to identify potential confounding factors and the sample size was adequately powered to adjust the effect of confounders while investigating the objectives of the study. On the contrary, it was a hospital based case-control study which limited the generalizability of the results only to patients visiting hospitals. We measured BMI at the time of calling patient for research. Although, BMI at time of presentation of disease does not always correlate with BMI after treatment. As case-control study design was implemented for this study, the responses were based on self-reported measures that can possibly lead to the presence of recall bias. This study provided evidence about the potential risk factors of breast cancer, especially considering Afghan women. Here is a dire need to conduct multi-center longitudinal cohort studies to explore temporal relationship between various factors and breast cancer.

## Conclusion

This study demonstrated that age, age at menarche, age at first baby’s birth, illiteracy, smoking and family history of cancer were the positive predictors of developing breast cancer among women in Afghanistan. Attempts should be made to educate women regarding the risk factors associated with development of breast cancers, in an attempt to reduce the prevalence and thus decrease the burden of breast cancer and its impact on the healthcare system in Afghanistan. It is needed that the data system in the hospitals of country must be improved so that these type of data are recorded at time of admission of patients.

## Data Availability

Data and materials of this work are available from the corresponding author on reasonable request.

## Abbreviations

IDC: Invasive ductal carcinoma
ILC: Invasive lobular carcinoma
BMI: Body mass index
WHO: World Health Organization
FMIC: French Medical Institute for Mothers and Children
JTCH: Jamhoriat tertiary care hospital
ITCH: Isteqlal tertiary care hospital
ILMS: Integrated laboratory management system
SD: Standard deviation
OR: Odds ratio
CI: Confidence interval
pSTAT5: Signal transducer and activator of transcription 5
JAK-STAT5: Janus kinase and signal transducer and activator of transcription 5
BRCA1: Breast Cancer gene 1
BRCA2: Breast Cancer gene 2
CHEK2: Checkpoint kinase 2
